# Clouds buffer Arctic warming

**DOI:** 10.1093/nsr/nwaf165

**Published:** 2025-04-25

**Authors:** Yuan Wang

**Affiliations:** Department of Earth System Science, Stanford University, USA

The Arctic climate system, a fully coupled climate system involving the atmosphere, ocean and cryosphere, is undergoing profound transformations. The snow and ice over the Arctic have been retreating at an alarming pace due to anthropogenic warming. Moreover, cloud feedback remains one of the most uncertain components in projections of Arctic climate change. Clouds play a dual radiative role, cooling the surface via the reflection of shortwave radiation and warming it through trapping the infrared radiation. The sign and magnitude of the cloud radiative effect partly depend on the surface albedo (SA), i.e. a dark surface can amplify the cloud radiative cooling. The highly reflective and rapidly changing surface of the Arctic makes the quantification of their net cloud impact rather challenging. This uncertainty is further compounded by sparse observations at the process level, complex cloud microphysical properties (e.g. phase partitioning between ice and liquid), and co-varying meteorological conditions. A new study [1] quantifies the feedback between snow/ice coverage (SIC), SA and the cloud radiative effect (CRE) over the Arctic across multiple temporal scales and observational-model platforms. It reveals that as SA declines in the Arctic, cloud shortwave cooling effects intensify, partially mitigating further snow and ice melting.

With their long-term, wide-coverage measurements of atmospheric radiations, cloud properties and SIC, satellite observations remain a powerful research tool for assessing Arctic climate change and its related physical processes. The new analysis [1] leverages the 20-year records (2000–2020) of NASA satellites (MODIS and CERES) to establish a grid-based linear relationship between the Arctic SIC and SA on both seasonal and interannual timescales. The finding that a 1% decline in SIC results in a ∼0.57% decrease in SA reinforces the pivotal role of snow/ice feedback in shaping Arctic energy balance. The observed relationships are also used to evaluate the state-of-the-art climate model intercomparison project phase 6 (CMIP6) model historical simulations.

The new study further investigates how changes in SIC translate into changes in CRE at both the top of the atmosphere (TOA) and surface (SFC). Notably, during the past two decades, the decline in SIC (∼0.058/decade) has contributed to a cooling CRE trend of −1.25 ± 0.49 W/m²/decade at TOA and −0.21 ± 0.20 W/m²/decade at the surface. This enhanced shortwave cooling effect by clouds is estimated to slow sea ice melt by up to 3.45 cm/year, a significant climate buffering mechanism. Importantly, this feedback appears seasonally modulated, peaking in June–July when insolation is highest. Moreover, by examining future projections by CMIP6 models, the analysis finds that cloud shortwave cooling becomes more pronounced under stronger warming scenarios. By extending the analysis through the 21st century under different shared socio-economic pathway (SSP) scenarios, the new study shows that CRE becomes increasingly negative as SIC decreases, suggesting an inherent compensatory mechanism embedded within the Arctic climate system.

While earlier research [2] has recognized the roles of clouds and SA in Arctic climate feedback, this study uniquely synthesizes these components by establishing a direct and quantifiable link between SIC and CRE changes. While clouds cannot reverse the course of sea ice melting, their role in moderating the pace of Arctic warming is important. As the Arctic marches toward a seasonally ice-free future, understanding both individual and total feedback is more critical than ever.

This study opens several avenues for further investigation. First, as cloud properties themselves may respond to SIC changes, the detailed feedback mechanisms remain to be explored. Future studies should focus on cloud microphysical responses (e.g. cloud phase, droplet size) to understand whether cloud radiative forcing may non-linearly evolve under continued Arctic warming. Note that the treatments of the Arctic clouds in the current global climate model (GCM) remain crude, due to the unresolved spatial structure of clouds at the subgrid scale and simplified microphysical schemes. Moreover, aerosol–cloud interactions add a further layer of complexity. Aerosols from mid-latitudes and local Arctic sources can modify cloud condensation nuclei (CCN) and ice-nucleating particle (INP) concentrations, altering cloud droplet number, size and lifetime [3]. The opened surface following ice/snow melting can potentially provide more precursor gases for local aerosol formation. Therefore, the role of aerosol in the SIC–CRE relationship identified by the new study would be an interesting research question to address.

Methodologically, while this study provides an observational-modeling consistency, it relies heavily on linear regression. Incorporating machine learning or non-linear analysis methods could uncover more complicated physics or thresholds in cloud responses and effects. Across the Arctic, some regional features of feedbacks deserve more attention. The strongest ∆CRE and ∆SIC values were observed in marginal seas (Beaufort, Chukchi, etc.), suggesting these areas are ‘hotspots’ of Arctic cloud-radiative feedback. Targeted field campaigns in these locations, such as MOSAiC (Multidisciplinary drifting Observatory for the Study of Arctic Climate), would further validate the inferred relationships.
